# Refractive Index Compensation in Over-Determined Interferometric Systems

**DOI:** 10.3390/s121014084

**Published:** 2012-10-19

**Authors:** Josef Lazar, Miroslava Holá, Ondřej Číp, Martin Čížek, Jan Hrabina, Zdeněk Buchta

**Affiliations:** Institute of Scientific Instruments, Academy of Sciences of the Czech Republic, Královopolská 147, 612 64 Brno, Czech Republic; E-Mails: hola@isibrno.cz (M.H.); ocip@isibrno.cz (O.Č.); cizek@isibrno.cz (M.Č.); shane@isibrno.cz (J.H.); buchta@isibrno.cz (Z.B.)

**Keywords:** refractometry, nanopositioning, interferometry, nanometrology

## Abstract

We present an interferometric technique based on a differential interferometry setup for measurement under atmospheric conditions. The key limiting factor in any interferometric dimensional measurement are fluctuations of the refractive index of air representing a dominating source of uncertainty when evaluated indirectly from the physical parameters of the atmosphere. Our proposal is based on the concept of an over-determined interferometric setup where a reference length is derived from a mechanical frame made from a material with a very low thermal coefficient. The technique allows one to track the variations of the refractive index of air on-line directly in the line of the measuring beam and to compensate for the fluctuations. The optical setup consists of three interferometers sharing the same beam path where two measure differentially the displacement while the third evaluates the changes in the measuring range, acting as a tracking refractometer. The principle is demonstrated in an experimental setup.

## 1. Introduction

Dimensional metrology on the fundamental metrology level is a domain of various interferometric techniques. It means counting (and interpolation) of single wavelengths of a coherent light source representing elementary quanta of length. This principle is consistent with the definition of length where the physical constant—speed of light—can be seen as a conversion from optical frequency into wavelength. Realization of the length standard is thus a highly stable laser source, stable in optical frequency. Under vacuum conditions the conversion to stable wavelength does not mean any loss in uncertainty.

The stability of the optical frequency of laser sources which has been achieved recently is very high. Traditional He-Ne lasers stabilized to the active Doppler-broadened line in Ne can operate with relative frequency stabilities at the 10^−8^–10^−9^ level, He-Ne lasers stabilized through subdoppler spectroscopy in iodine on the 10^−11^–10^−12^ level and the potential of iodine-stabilized lasers based on frequency doubled Nd:YAG is very close to the 10^−14^ level [1]. The reproducibility of their absolute frequencies is another goal in metrology and is limited to 2.1 × 10^−11^ and 9 × 10^−12^, respectively [2] and the absolute frequency value is primarily limited by the absorbing medium [3].

Dimensional measurement of real objects has to be mostly done on air, not only for practical reasons, but also due to the influence of atmospheric pressure on their size. Under atmospheric conditions the value of the refractive index of air has to be considered. The search and effort for a more precise interferometric measuring tool includes highly stable laser sources, reduction of noise, better optics, higher resolution through optical and electronic techniques, linearization, *etc.* [4–6]. Obviously, when measurement has to be performed in air—under laboratory measurement conditions or even in industrial applications—the refractive index of air represents a key source of uncertainty.

Direct and absolute measurement of the refractive index of air can be done by a laboratory refractometer. To get the precision needed it has to operate on an inverse principle than the length measuring interferometer. Mechanical length stays constant while the optical one is varying from the vacuum to atmospheric optical length [7–9]. Many variations of this principle have been presented [10–12]. Instruments designed to measure the refractive index on-line are called tracking refractometers; they should complement the most precise length measurements [13]. A tracking refractometer converting the refractive index variations into laser optical frequency has been presented in [14]. In this case the concept relies on coherent and broadly tunable laser sources [15,16].

Any measurement of the refractive index of air via a laboratory refractometer or its indirect evaluation through the Edlen formula [17] is limited by the fact that air is an inhomogeneous medium. There are always thermal gradients present in the air—mainly in the vertical direction and air flow, especially on the microscopic scale, cannot be completely eliminated. There will always be a difference between the measuring beam line and the place of measurement of the refractive index, not to mention its varying value along the measuring axis. However special laboratory measurements of the refractive index can be done with an uncertainty close to the 10^−9^ [8,18]. The most precise laboratory techniques seem to be those exploiting optical frequency comb synthesis [19–21], similar to those where the length is directly measured with the help of an optical synthesizer [22,23].

Evaluation of the refractive index using the Edlen formula is based on measurement of the fundamental atmospheric parameters—temperature, pressure and humidity of air, accompanied in some cases by measurements of carbon dioxide concentration. The refractive index of air is only approximated so improved, more precise versions have been published [24–27]. A lot of effort has been put into evaluation of other effects such as content of various gases in air, especially CO_2_ [28]. Humidity, particularly the content of water vapor, has been investigated in [29,30]. The study of these effects resulted in inverse approaches where measurement of the refractive index of air became the means of determining another quantity, such as temperature [31] or air density and moisture [32,33].

**Figure 1.**
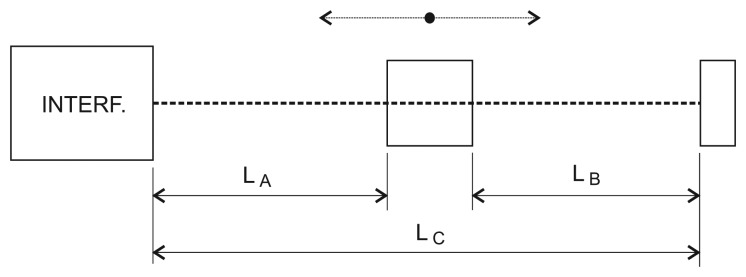
Principal schematics of the inteferometric system with two countermeasuring interferometers and an interferometer monitoring overall length L_C_. L_A_, L_B_: particular lengths determining the position of the moving carriage.

Efforts to combine the distance measuring interferometer and the refractometer into one instrument which could evaluate the influence of the refractive index of air during the measurement or directly compensate for it have been reported. There were arrangements presented where a complex set of two separate interferometers evaluates the refractive index of air and measures the distance [34]. This system can compensate the refractive index, but is unable to overcome the problem of the determination of the refractive index in the laser beam axis. A method linking the wavelength of the laser source to the mechanical length of some frame or board was proposed in [35]. The authors suggested using a set of two identical interferometers where one is fixed in the length and serves as a reference for the laser wavelength. Other approaches represent completely different methods for determination of the refractive index of air, for example through the speed of sound in the ultrasonic frequency range [36]. Also, the control of the refractive index which is kept constant was suggested [37]. We proposed a concept with an over-determined counter-measuring interferometric displacement measuring setup [38–40] where the length in one axis was measured by two interferometers with their position fixed to a highly stable mechanical reference. In this contribution we present a new version of this concept with three interferometers with corner-cube reflectors where the overall length is not a sum value of two, but rather an independently measured value (Figure 1).

## 2. Experimental Configuration

In this proposed concept the reference relied on a material with thermal stability low enough to overcome the uncertainty caused by fluctuations of the refractive index of air. We used “0”–grade Zerodur ceramics from Schott, with stability at the 10^−8^/K level for a wide range of temperatures from 0 °C to 50 °C. In a smaller range the coefficient of thermal expansion should have a plateau with even smaller thermal expansion.

In our concept the wavelength of the laser source was fixed by a control loop to a sum value of the two interferometers representing a principle of stabilization of wavelength instead of traditional stabilization of laser optical frequency and compensation of fluctuations of the refractive index of air by indirect measurement. In this experiment we have tested the principle using two plane-mirror interferometers. The alignment of the system, very sensitive to any angle deviation and cosine errors proved to be very difficult. It could hardly be used for real displacement measurements due to the extreme sensitivity to the tilt of the moving mirror. The experiment presented in [38] thus had to be done in a static regime.

**Figure 2.**
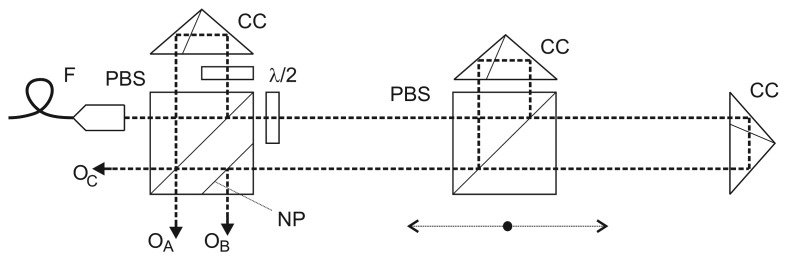
Configuration with corner-cube reflectors measuring directly the overall length and two particular displacements. CC: corner-cube reflector, PBS: polarizing beamsplitter, NP: non-polarizing plane, λ/2: half-wave plate, F: fiber-optic light delivery, O_A_, O_B_, O_C_: outputs.

The design of this arrangement was motivated by an effort to come up with a concept suitable for metrological applications, especially in nanometrology where the positioning is done within a specified range, the setup is fixed to a “metrology frame” made of a stable material and the measurement has to be performed in air. We applied a more traditional approach with corner-cube reflectors thus eliminating the errors caused by angle deviation of the moving carriage. The system consists of three independent interferometers where each measures a specified part of the overall length (A, B, C, see Figure 1). All three interferometers are using polarization-separated reference and measuring arms and homodyne detection. The operating wavelength is λ = 532 nm, and they are fed by a high-stability and low-noise, metrology-grade frequency-doubled Nd:YAG laser. The interference signal is digitized (12-bit DACs) and processed in a DSP unit with fringe counting embedded in the hardware and the actual phase is calculated in a DSP processor. The hardware as well as the software have been developed at our Institute. The processing of the interference signal includes a linearization method [41,42]. Linearity errors of the interferometers were at the 3–4 nm level. After the evaluation of the interference phase the wavelength resolution of λ/2,048 results in a resolution of 260 pm for 533 nm wavelength. The linearization technique implemented here reduces the linearity error to the level of a single discrete LSB (least significant bit)—the resolution of the interferometer. Similar techniques have been presented by other groups and are widely used in various versions in nanometrology [43–45]. The optical arrangement is shown in Figure 2. The left polarizing beamsplitter with a corner-cube reflector serves as a reference arm for the interferometer measuring the distance between the left reference point and the moving carriage (A) as well as for the interferometer measuring the overall length (C). The moving carriage carries another beamsplitter with corner-cube reflector generating a reference arm for the interferometer measuring the distance between the moving carriage (B) and the right reference point. The beam of the interferometer C only passes through the beamsplitter on the moving carriage. Beam paths on air of the interferometers A and B are identical with proportional parts of the beam path of the interferometer C. The polarization plane of the input laser beam is oriented to get the power ratio on the left beamsplitter 1:2 to keep an equal amount of power for all three interferometers. The left beamsplitter is also equipped with an additional non-polarizing beamsplitting plane for separation of the output from the interferometer B.

This concept combines the principle of one-axis interferometric measurement with a Michelson type interferometer and a tracking refractometer that is able to follow the variations of the refractive index just in the beam path of the measuring interferometer. Our arrangement includes two interferometers measuring the displacement in a counter-measuring setup. This can be seen as an overdetermined interferometer where the position of the carriage may be referenced either to the left or right end of the measuring range. Still the identity of the displacement measuring beam path (on air) and the beam path of the tracking refractometer is limited by the ratio given by the carriage position. The value of the refractive index may differ in the left and right part (A, resp. B) of the setup. The best approximation of the resulting carriage position should be thus a value calculated from both A and B.

## 3. System Performance

The interferometric system was placed in a double-wall glass box with the walls filled with water. Circulation of the water with a pump ensured an even distribution of temperature on the walls and reduction of thermal gradients in air inside. The circulating water went through a power Peltier heater/cooler. This allows us to control the temperature inside and let the air be heated or cooled gradually so the refractive index of air would vary within some range. To monitor the atmosphere inside, we added temperature, pressure, and humidity sensors together with a sensor monitoring the content of CO_2_. Refractive index of air was instantly calculated and recorded from these measurements to be compared with the interferometer values.

**Figure 3.**
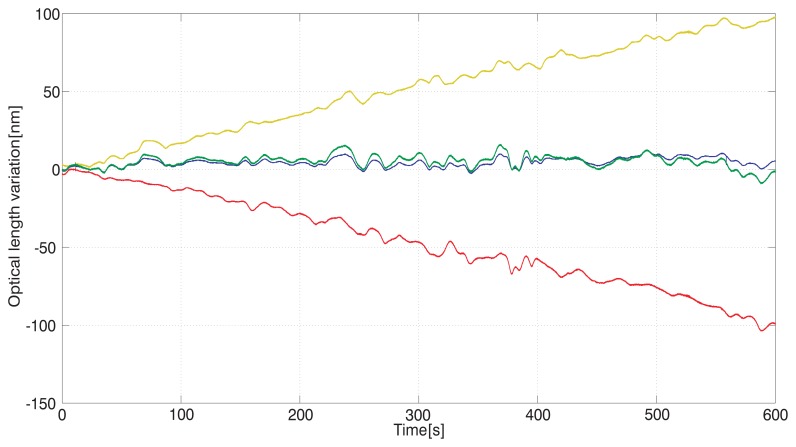
Recording of the variations of the interferometers A (red line), B (yellow line), and overall length measuring C (blue line) together with the sum of A and B (green line) over time in a closed thermal box.

The recording in Figure 3 shows outputs from the three interferometers (A, B, and C) when the temperature controlling box was closed and the air flow reduced to minimum (convection air currents inside).

Outputs from the interferometers were recorded with their counters reset at the start of the measurement. The carriage was approximately in the middle of the measuring range. The air path of the whole measuring range (monitored by the interferometer C) was 195 mm. The recording shows absolute changes of the measured optical lengths over the time interval 10 min. There is also a small and slow mechanical drift of the carriage position in one direction superimposed on the outputs of the interferometers A and B resulting in increasing of the output of one and decreasing of the output of the other. We also add the sum of A and B. This value shows a good agreement with the result of the interferometer C showing that the output C can monitor the varying refractive index in both A and B very well.

**Figure 4.**
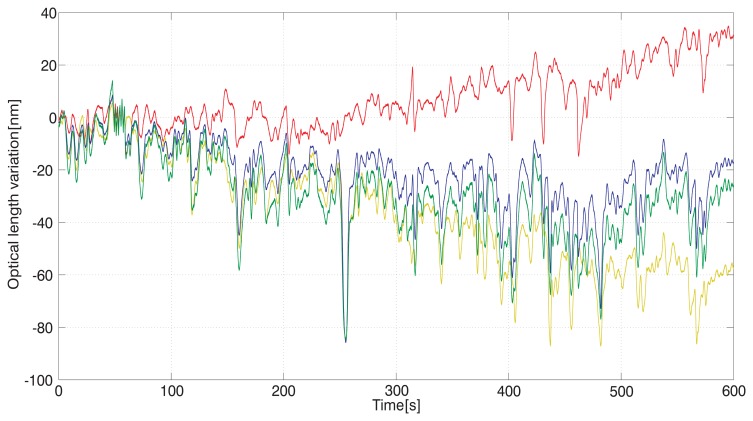
Recording of the variations of the interferometers A (red line), B (yellow line), and overall length measuring C (blue line) together with the sum of A and B (green line) over time in under laboratory environment.

To demonstrate the influence of air flow we recorded the fluctuations of the interferometers' output following the variations of the refractive index of air with the temperature controlling box opened. The setup was subject to the laboratory conditions still with no major source of air circulation. The recording of the three interferometers output is shown in Figure 4 together with a sum value of A and B to be compared with the overall optical length C.

**Figure 5.**
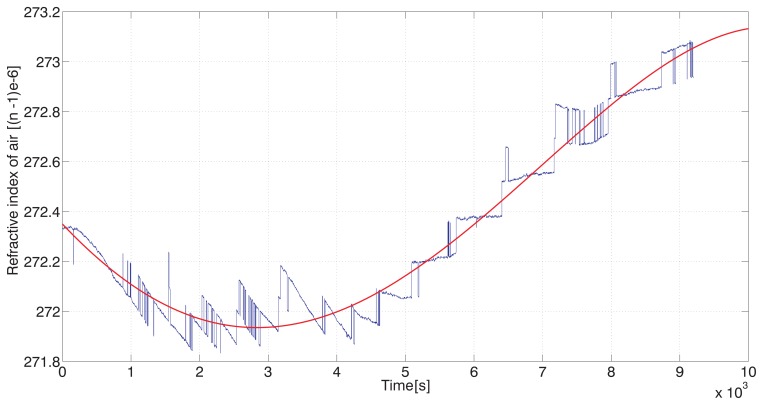
Recording of a slow refractive index drift evaluated from measurement of air temperature, pressure, humidity and CO_2_ content (blue line) and polynomial approximation (red line).

To test the ability of the system to follow the drift of the refractive index of air, we recorded the interferometers' values during heating of the air inside of the thermal box. Again, the water flow in the walls reduced thermal gradients inside and the heating of the inner air was uniformly distributed. Recording of the refractive index drift evaluated indirectly from the physical parameters of atmosphere is given in Figure 5. There are visible steps caused by a limited resolution of the CO_2_ content sensor. To get a diagram representing the drift we applied a polynomial approximation of the recorded data smoothing the refractive index variations. The recording can be interpreted as a slow drift from one steady state to another.

**Figure 6.**
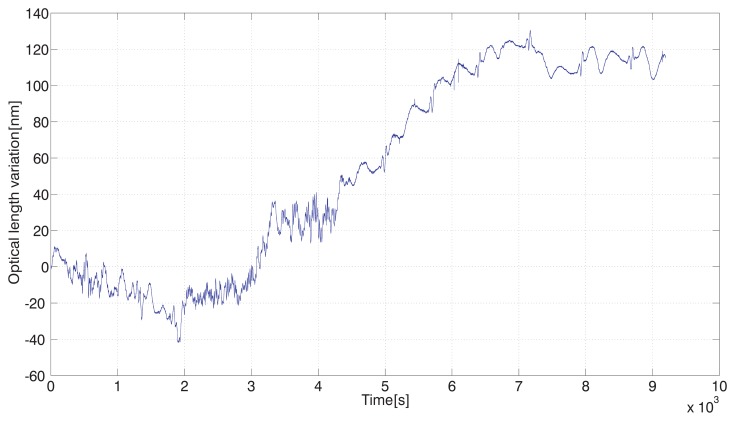
Recording of the output from the interferometer C, responding to variations of the overall optical length.

The simultaneous recording to be compared is an output from the interferometer C, measuring variations of the absolute optical length in nm. Fast small-scale variations of the interferometer C output (Figure 6) compared to the whole recorded drift show how interferometric measurement is influenced by the atmosphere, even in a closed environment. Indirect evaluation of the refractive index as shown in Figure 5 (not mentioning the CO_2_ content steps) is unable to follow these fluctuations due to the slow response of the sensors.

The traces in Figures 5 and 6 obtained during heating show a “phase shift” caused by a slow response of the sensors used for measuring the atmospheric parameters. Then there is a gradual change of optical frequency drift while the refractive index still rises. To follow the principle of referencing to high-stability mechanical frame, it should include the central beamsplitter on the moving carriage to be made out such a material as well, at least quartz glass. In our case we used SF-14 glass for technology reasons and its slow gradual heating together with high thermal expansion coefficient and high refractive index consequently acted against the course of the drift. A good agreement can be found only in the beginning of the temperature (refractive index) rise within a period of approx. 30 min before the expansion of the glass showed up. This agreement is on the 4 × 10^−8^ level of the refractive index change and related relative optical length change.

## 4. Discussion and Conclusions

The presented arrangement tests the ability of the interferometric system to follow the fluctuations of the refractive index within a measuring range given by the interferometer measuring the overall length, “C”. Recordings made under steady conditions with non-varying temperature in a closed box and under laboratory environment conditions show the level and character of the refractive index of air fluctuations. They also provide information about the level of agreement between the particular paths, here referred to as “A” and “B” and representing the displacement of the carriage. This may be seen as a limiting factor of the resolution and of the possibilities of the method to compensate for the drift. Fluctuations of A, B, and C are expressed in Figures 3 and 4 as absolute changes of the optical lengths. The level of agreement between them shows that the best approximation of the measured carriage displacement should be derived from both A and B values, most likely an average of both.

To compensate fluctuations of the refractive index of air the output of the interferometer C should be considered as a tracking refractometer. Its key advantage is a measurement of these variations in the beam path of both displacement measuring interferometers. This advantage can be expressed as the level of agreement mentioned above. Under closed box conditions this value is less than 5 nm for an overall air path of 195 mm. In relative terms this is equal to 2.5 × 10^−8^. To calculate compensated values of the A, resp. B position, the relative variations of C can be used with the proportion to the absolute length of A, resp. B:

(1) $$A_{\text{comp}.}=\text{A}-\text{A}\frac{\Delta C}{C}$$

(2) $$B_{\text{comp}.}=B-B\frac{\Delta C}{C}$$

where ΔC represents actual absolute variation of the optical length measured by the interferometer C and A*_comp_*., resp. B*_comp_*. represent compensated (corrected) values of A and B. Incremental interferometry, a technique applied here, is able to measure precisely only displacement, a change of absolute length when interference fringes are continuously counted. The absolute lengths (absolute position of the carriage) A and B as well as the overall length C used to calculate the compensation do not have to be known with precision down to nm level. Relative uncertainty of the A, B and C values used for this calculation project themselves into the uncertainty of the corrective increment for A, resp. B. For example if A = 100 mm, the fluctuation of C due to the varying refractive index is 10 nm, the uncertainty of the absolute length of A at 1 mm level results in an error of the increment used to compensate for this fluctuation on the order of 10^−9^.

On the one hand there is a good agreement between the long-term drift of the refractive index measured and that evaluated through the Edlen formula and the recording made by our tracking refractometer. On the other hand the major difference is on the scale of seconds and tens of seconds. As stated before, the system cannot completely bypass the indirect evaluation of the refractive index. It is able to follow only the fluctuations. Relying on a high stability material such as Zerodur and the need to compensate even the smallest material thermal drift may make it too expensive and complicated, but when seeking improvements on the scale where the differences are (seconds and tens of seconds) the slow thermal expansion of the material may not be the crucial problem. The measurement can still rely on the Edlen formula for slow monitoring of the refractive index and on the scale below approx. 1 min limit this method can be included into the measurement scheme. Thus, even with no expensive low-expansion materials it can bring about an improvement. This improvement may be seen as a sort of noise-eater for noise introduced by fast-varying fluctuations of the atmosphere.

The concept presented here and the experimental arrangement built to prove it show that it may contribute to practical interferometry and may even find its way into technical practice. Its main field of application seems to be metrological nanocomparators where the measuring range is limited to several cm and the systems have to be operated in air. Potentially, also in long-range coordinate measuring schemes for nanometrology, where this concept has to be modified for two- or more axis measurement configuration. The evaluation principle where the overall length measuring interferometer serves as a tracking refractometer is useful for fixed-frequency lasers or two-frequency lasers suitable for heterodyne interferometry. Lasers with a fast-tuning option may be operated in a wavelength stabilization regime resulting in a sort of standing wave interferometer. These are the challenges for the future.
